# Use of Systemic Anticoagulation in COVID-19: Delving Beyond Theoretical Hypothesis

**DOI:** 10.7759/cureus.22061

**Published:** 2022-02-09

**Authors:** Christopher Millet, Spandana Narvaneni, Fady Shafeek, Sherif Roman, Ashesha Mechineni, Rajapriya Manickam

**Affiliations:** 1 Internal Medicine, St. Joseph's Regional Medical Center, Paterson, USA; 2 Pulmonary and Critical Care Medicine, St. Joseph's Regional Medical Center, Paterson, USA

**Keywords:** covid-19 disease, covid-19 management, stroke and covid-19, covid coagulopathy, dvt prophylaxis, pulmonary embolism (pe), venous thromboembolism (vte), systemic anticoagulation, covid-19

## Abstract

Background

Studies suggest that COVID-19 infection may induce increased hypercoagulability, leading to thrombotic complications. The high rates of thrombotic complications among patients receiving standard-dose deep venous thrombosis (DVT) prophylaxis have prompted some clinicians to support the empiric increase of anticoagulation (AC) doses used for prophylaxis in patients with COVID-19. At present, the optimal anticoagulant agents, dosages, and duration have not been designated. We conducted a retrospective study to assess for outcomes in patients who received treatment for COVID-19 based on various dosings of AC.

Methods

This was a single-institution, retrospective cross-sectional study including patients with a positive COVID-19 test who were admitted within the St. Joseph’s Health Network from September to November of 2020. The inclusion criteria were men and women aged 18 years or older who had confirmed COVID-19 by polymerase chain reaction (PCR). Medical charts of patients who met the inclusion criteria were audited to obtain information. The patients were separated into three cohorts: those who received DVT prophylactic dose of AC, those who received an intermediate dose of AC, and those who received therapeutic AC.

Results

A total of 440 patients were included in the study, of whom 236 were Hispanic (50.3%), 131 were Caucasian (27.1%), 47 were African American (10.7%), and 26 were Asian (5.9%). The most common comorbidities were hypertension (273/440 [62.2%]), diabetes 189/440 [43.1%]), and coronary artery disease (60/440 [13.7%]). In the DVT prophylactic dose of AC cohort, there were 215 patients, and the average length of stay was 10.3 days. Eleven patients experienced bleeding events, five patients experienced thrombotic events, 16 patients required mechanical ventilation, and 20 patients died. In the intermediate dose of AC cohort, there were 63 patients, and the average length of stay was 10.3 days. Three patients experienced bleeding events, two patients experienced thrombotic events, seven patients required mechanical invasive ventilation, and 11 patients died. In the therapeutic dose of AC cohort, there were 162 patients, and the average length of stay was 14 days. In this cohort, 19 patients experienced bleeding events, 12 patients experienced thrombotic events, 26 patients required invasive mechanical ventilation, and 29 patients died. Patients who received intermediate dosing of AC also had the lowest risk of thrombotic events (0.05). Patients who received intermediate dosing of AC had the lowest rates of requiring both high-flow nasal cannula (p = 0.0001) and invasive mechanical ventilation (p = 0.031). Patients who received intermediate dosing of AC had a lower rate of bleeding compared to those who received the DVT prophylaxis dose and systemic AC dose (p = 0.037). The DVT prophylactic and intermediate dosing of AC groups had a shorter length of stay in comparison to the systemic AC group (p = 0.0002).

Conclusion

In comparison to the venous thromboembolism prophylaxis dose and systemic AC dose groups, intermediate dosing of AC had the lowest rates of hemorrhage, mortality, length of stay, and requirement of high-flow nasal cannula or mechanical invasive ventilation. In the systemic dose AC group, there were worse clinical outcomes in terms of length of stay, incidence of bleeding events, requirement of mechanical ventilator use, and rate of mortality.

## Introduction

In December of 2019, the coronavirus disease 2019 (COVID-19) was first identified in Wuhan, China. Soon thereafter, the virus spread to become a global pandemic, with a devastating impact on communities and the world economy. Multiple therapeutic interventions for COVID-19 were established; however, most of the utilized therapeutic agents did not show clinical benefit or decreased mortality. Due to the worldwide crisis, the guidelines for COVID-19 management were made under conditions of uncertainty, leading to many variations in recommendations and a lack of inclusivity [1].

The evolution of our understanding of the pathogenesis of COVID-19 has had a significant impact on the development of management strategies. Several clinical trials have since assessed the efficacy of multiple medications used in the early phase of the pandemic for treating COVID-19. For example, one study found that hydroxychloroquine, whether alone or in combination, was not effective in treatment of COVID-19 and found to have additional safety concerns related to its use [2]. Moreover, multiple trials were conducted to assess the possible benefits of therapies that block IL-6, IL-1β, TNF-α, and IL-1, which revealed no clear evidence to support mortality reduction with their use [3]. Additionally, high-dose intravenous immunoglobulin (IV Ig) has been tested, and studies revealed that it may have a beneficial effect in the hyperinflammatory state, but the mechanism of action is unknown [4]. Also, several nutritional components and vitamins such as vitamin C, zinc, and vitamin D may have theoretical but unproven clinical benefits yet. Furthermore, multiple antiviral agents such as remdesivir are being studied in various clinical trials as well [5]. Corticosteroids for the treatment of COVID-19 have shown promising results in patients requiring oxygen support. According to the RECOVERY trial (Randomized Evaluation of COVID Therapy), the use of corticosteroids decreased the risk of mortality, duration of hospital stay, and need for mechanical ventilation [6].

Although respiratory compromise is the disease's cardinal characteristic, many reports suggest increased thrombotic complications associated with COVID-19 infection. Studies suggest that the hyperinflammatory condition in COVID-19 induces endothelial cells injury and increases hypercoagulability, which can lead to thrombotic complications [7]. Also, the hypoxia found in severe COVID‐19 can lead to thrombosis by increasing blood viscosity and a hypoxia‐inducible transcription factor‐dependent pathway [8]. The high rates of these thrombotic complications even among patients receiving standard-dose thromboprophylaxis have prompted some clinicians to support the empiric increase of anticoagulation doses used for prophylaxis in patients with COVID-19 [9].

While several major societies have supported standard-dose thromboprophylaxis, some studies recommend continuing enoxaparin for 7 to 14 days after patients return to their normal daily activities due to persistent hypercoagulopathy after the acute phase of the disease [7]. Currently, there is inadequate high-quality data for evidence-based decision-making, and most current recommendations are derived from descriptive studies. At present, the optimal anticoagulant agents, dosages, and duration have not been designated, and high-quality clinical trials are required. We conducted a retrospective study to assess for outcomes in patients who received treatment for COVID-19 based on various dosing of anticoagulation.

## Materials and methods

Study design

This was a retrospective cross-sectional study that was conducted at St. Joseph’s University Hospital. The study was reviewed and approved by the Institutional Review Board of St. Joseph’s University Hospital (IRB approval #00000892). Patients included in the study were those with a positive COVID-19 test who were admitted to a hospital within the St. Joseph’s Health Network from September to November of 2020. Data were obtained from auditing medical charts during the month of February of 2021.

Study population

This was a single-institutional study consisting of COVID-19 positive patients admitted within the St. Joseph’s Health Network. The two hospitals comprising this network include St. Joseph’s University Hospital in Paterson, New Jersey, and St. Joseph’s Hospital in Wayne, New Jersey. The inclusion criteria were men and women aged 18 years or older who had confirmed COVID-19 by polymerase chain reaction (PCR). Patients who were excluded from the study were patients who did not have a confirmed COVID-19 test using PCR testing and were not admitted during the months of September to November of 2020.

Study procedures

A list from the medical records was generated, which consisted of patients admitted to the hospitals within the months of September and November who tested positive for COVID-19. Medical charts of patients who met the inclusion criteria were then audited, and information was collected, including labs, medications and therapies provided, and clinical course, as well as baseline and demographic information. The patients were separated into three cohorts: those who received DVT prophylactic dose of anticoagulation, those who received intermediate anticoagulation, and those who received therapeutic anticoagulation. Intermediate dose was defined as any dose of anticoagulation used for patients higher than prophylactic dose but not a systemic dose. After data collection was complete, data underwent statistical analysis using chi-square analysis.

## Results

There were 440 patients admitted with a positive COVID-19 test who met the criteria to be enrolled in the study (Table 1). Of the 440 patients included in the study, 236 were Hispanic (50.3%), 131 were Caucasian (27.1%), 47 were African American (10.7%), and 26 were Asian (5.9%). The most common comorbidities were hypertension (273/440 [62.2%]), diabetes 189/440 [43.1%]), and coronary artery disease (60/440 [13.7%]). The patients were separated into three cohorts: those who received a prophylactic dose of enoxaparin or heparin, those who received an intermediate dose of anticoagulation, and those who received a therapeutic dose of anticoagulation (Table 1). Among the cohort who received DVT prophylactic dose of anticoagulation, some of the baseline characteristics were as follows. There were 215 patients, which accounted for 48.9% of the entire study population. The average age was 60.86 years, the population was composed of 146 males and 89 females, and the average length of stay was 10.3 days. There were 11 patients who experienced bleeding events and five patients who experienced thrombotic events. There were 16 patients who required mechanical ventilation, and 20 patients died (Table 2). Among the cohort who received an intermediate dose of anticoagulation, some of the baseline characteristics were as follows. There were 63, patients which accounted for 14.3% of the entire study population. The average age was 63 years, and the cohort consisted of 35 males and 28 females. The average length of stay was 10.3 days. There were three patients who experienced bleeding events and two patients who experienced thrombotic events. There were seven patients who required mechanical invasive ventilation, and 11 patients died (Table 2). Among the cohort who received a therapeutic dose of anticoagulation, some of the baseline characteristics were as follows. There were 162 patients, and the average age was 91 years in males and 74 years in females. The average length of stay was 14 days. There were 19 patients who experienced bleeding events and 12 patients who experienced thrombotic events. There were 26 patients who required invasive mechanical ventilation, and 29 patients died (Table 2).

**Table 1:** Demographic and baseline characteristics comparing populations based on systemic anticoagulation treatment received during hospitalization HFrEF, heart failure with reduced ejection fraction; HFpEF, heart failure with preserved ejection fraction; CKD, chronic kidney disease; ESRD, end-stage renal disease

Variable	Prophylactic dose, N=215	Intermediate dose, N=63	Therapeutic dose, N=162	p-Value of comparison
Age	60.86 (16.29)	64.25 (14.76)	65.89 (12.5)	0.004
Male	146 (68%)	35 (55.5%)	91 (56.2%)	0.037
Ethnicity groups				
- White/Caucasian	56 (26%)	19 (30%)	44 (27.2%)	0.92
- Hispanic/Latino	111 (51.6%)	31 (49.2%)	80 (49.4%)	
- Black/African American	21 (9.8%)	7 (11.1%)	19 (11.7%)	
- American Asian	16 (7.4%)	2 (3.2%)	8 (5%)	
- Other	11 (5.1%)	4 (6.3%)	11 (6.8%)	
Medical comorbidities				
Coronary artery disease	22 (10.2%)	5 (7.9%)	33 (20.4%)	0.008
Atrial fibrillation/flutter	3 (1.4%)	1 (1.6%)	26 (16%)	0.0001
Peripheral vascular disease	2 (0.9%)	1 (1.6%)	5 (3%)	0.3
Diabetes	84 (39%)	28 (44.4%)	78 (48.1%)	0.21
Congestive heart failure				
- HFrEF	7 (3.3%)	2 (3.2%)	10 (6.2%)	0.31
- HFrEF <30%	5 (2.3%)	0	2 (1.2%)	
- HFpEF	9 (4.2%)	2 (3.2%)	14 (8.6%)	
Asthma	9 (4.2%)	7 (11.1%)	21 (12.9%)	0.007
Chronic obstructive pulmonary disease	12 (5.6%)	2 (3.2%)	11 (6.8%)	0.57
Bleeding disorder/history of bleed	4 (1.8%)	0	6 (3.7%)	0.21
Alcohol abuse	6 (2.8%)	1 (1.6%)	5 (3%)	0.82
Obstructive sleep apnea	13 (6%)	1 (1.6%)	5 (3%)	0.19
Malignancy	10 (4.7%)	4 (6.3%)	6 (3.7%)	0.69
Prior cerebral vascular accident	12 (5.6%)	0	9 (5.5%)	0.16
Chronic kidney disease				
- CKD I-II	0	1 (1.6%)	5 (3%)	0.25
- CKD III-IV	15 (7%)	4 (6.3%)	12 (7.4%)	
- CKD V/ESRD	8 (3.7%)	2 (3.2%)	9 (5.5%)	

**Table 2:** Inpatient characteristics of the three different groups and comparison HgbA1c, hemoglobin A1c; LDH, lactate dehydrogenase; CRP, c-reactive protein; PT, prothrombin time; PTT, partial thromboplastin time; INR, international normalized ratio; PLT, platelet; BiPAP, bilevel positive airway pressure; WHO, World Health Organization

Variable	Prophylactic dose, N=215	Intermediate dose, N=63	Therapeutic dose, N=162	p-Value of comparison
Symptom onset to presentation	6.64 (3.83)	5.71 (3.08)	6.28 (4.11)	0.219
Pneumonia on imaging	171 (79.5%)	59 (93.6%)	144 (88.9%)	0.005
Acute kidney injury	73 (33.4%)	22 (35%)	68 (42%)	0.260
Liver disease	2 (0.9%)	2 (3%)	6 (3.7%)	0.176
New dialysis	3 (1.4%)	3 (4.7%)	5 (3%)	0.269
Septic shock	13 (6%)	6 (9.5%)	21 (13%)	0.068
Cardiac arrest	18 (8.4%)	11 (17.5%)	23 (14.2%)	0.072
Laboratory findings				
Peak ferritin (ng/mL)	819 (823)	932 (1301)	1091 (1646)	0.122
Peak troponin (ng/mL)	0.57 (3.65)	0.14 (0.43)	0.24 (1.36)	0.374
HgbA1c (%)	7.84 (2.33)	7.53 (2.1)	8.35 (2.36)	0.35
Peak LDH (units/L)	390.4 (186.75)	442( 314.4)	146 (311.61)	0.023
Peak CRP (mg/L)	123.7 (79.2)	142.9 (110.6)	144.05 (90.7)	0.071
Peak D dimer (mcg/mL)	3.62 (8.5)	5.45 (13.5)	4.07 (5.15)	0.34
Admission PT (seconds)	14.1 (1.37)	13.6 (2.53)	15 (4.93)	0.045
Admission PTT (seconds)	32.7 (6.58)	31.8 (5.3)	33.4 (9.28)	0.62
Admission INR	1.3 (2.88)	1.08 (0.1)	1.2 (0.62)	0.82
Lowest fibrinogen value ( )	453.4 (244.8)	467.7 (282.5)	454.4 (174.6)	0.993
Admission PLT (K/mm^3^)	218.83 (73.13)	231.2 (95.7)	232.98 (83.0)	0.211
Peak procalcitonin (ng/mL)	0.87 (3.9)	0.64 (1.7)	1.53 (5.25)	0.465
Medications				
Anticoagulation use at home	7 (3.3%)	1 (1.6%)	32 (19.7%)	0.0001
Antiplatelet use at home				
- Single	32 (14.9%)	8 (12.7%)	29 (46.8%)	0.806
- Dual	9 (4.2%)	2 (3.2%)	8 (5%)	
Inpatient antiplatelet therapy				
- Aspirin only	52 (24.2%)	10 (15.9%)	28 (17.3%)	
- Plavix only	4 (1.9%)	1 (1.6%)	7 (11.3%)	0.363
- Other agent only	149 (69.3%)	50 (79.4%)	118 (72.8%)	
- Dual antiplatelet therapy	10 (4.6%)	2 (3.2%)	9 (5.5%)	
Steroid use	186 (86.5%)	62 (98.4%)	155 (95.7%)	0.001
Remdesivir	164 (76.3%)	52 (82.5%)	127 (78.4%)	0.565
Tocilizumab	2 (0.9%)	0	1 (1.6%)	0.727
Plasma use	46 (21.4%)	18 (28.6%)	44 (27.2%)	0.316
Incidence of thrombotic events	5 (2.3%)	2 (3.2%)	12 (7.4%)	0.050
Incidence of bleeding events	11 (5.1%)	3 (4.8%)	19 (11.7%)	0.037
Mode of oxygen delivery				
- Nasal cannula	169 (78.6%)	56 (88.9%)	131 (80.9%)	0.189
- BiPAP	21 (9.8%)	7 (11.1%)	23 (14.2%)	0.409
- High-flow nasal cannula	43 (20%)	14 (22.2%)	101 (62.3%)	0.0001
- Ventilator	16 (7.4%)	7 (11.1%)	26 (16%)	0.031
WHO category				
- WHO category 3	34 (16%)	3 (4.8%)	13 (8%)	
- WHO category 4	119 (55.3%)	36 (57.1%)	68 (42%)	
- WHO category 5	39 (18.1%)	12 (19%)	48 (77.4%)	0.007
- WHO category 6	2 (0.9%)	1 (1.6%)	3 (1.8%)	
- WHO category 7	1 (0.4%)	0	1 (0.6%)	
- WHO category 8	20 (9.3%)	11 (17.5%)	29 (18%)	

The chi-square test was applied to compare variables among the three cohort groups. In comparison to the systemic anticoagulation group, the DVT prophylactic and intermediate dosing of anticoagulation groups had a shorter length of stay (p = 0.0002). Patients who received intermediate dosing of anticoagulation had a lower rate of bleeding compared to those who received DVT prophylaxis dose and systemic anticoagulation dose (p = 0.037). Patients who received intermediate dosing of anticoagulation also had the lowest risk of thrombotic events (p = 0.05). Patients who received intermediate dosing of anticoagulation had the lowest rates of requiring both high-flow nasal cannula (p = 0.0001) and invasive mechanical ventilation (p = 0.031) (Table 3).

**Table 3:** Final outcomes measured WHO, World Health Organization

Final outcomes	Prophylactic dose, N=215	Intermediate dose, N=63	Therapeutic dose, N=162	p-Value of comparison
Time for improvement of oxygenation by one WHO category in days, mean (SD)	4.43 (1.32)	4.87 (1.54)	4.99 (1.55)	0.008
Deceased, n (%)	18 (8.4%)	9 (14.3%)	24 (14.8%)	0.124
Length of stay in days, mean (SD)	10.3 (11.97)	10.3 (8.57)	14.5 (13.55)	0.002

## Discussion

In our study, patients received three varying doses of anticoagulation, including venous thromboembolism (VTE) prophylaxis dose, intermediate dose, and full-dose systemic anticoagulation. In our patient population, intermediate dosing of anticoagulation provided the best patient outcomes overall. In comparison to the VTE prophylaxis dose and systemic anticoagulation dose groups, intermediate dosing of anticoagulation provided the lowest rates of hemorrhage, mortality, length of stay, and requirement of high-flow nasal cannula or mechanical invasive ventilation. In the systemic dose anticoagulation group, there were worse clinical outcomes in terms of length of stay, incidence of bleeding events, requirement of mechanical ventilator use, and rate of mortality. Comparison of the groups showed comparable baseline characteristics except for age, gender distribution, and presence of cardiac comorbid conditions. All groups were comparable in terms of symptom onset to presentation, all other comorbidities, serum inflammatory markers, oxygen use requirements, and use of other therapeutic agents. Multiple studies have been conducted to determine the optimal treatment for hypercoagulability in patients with COVID-19. The outcomes of these studies have provided variable findings; thus, an optimal treatment protocol for hypercoagulation in COVID-19 remains undefined. We will now review the outcomes of these major studies as well as some of the current guidelines put forth by major societies on the utilization of anticoagulation in the treatment of patients with COVID-19 infection.

In 2021, a multiplatform, controlled trial consisting of 2,219 patients, which included data from the ATTACC, ACTIV-4a, and REMAP-CAP trials, was conducted to compare outcomes among hospitalized patients with COVID-19 who received different dosing of heparin. The study found that hospitalized patients who had moderate COVID-19 and were treated with full-dose anticoagulation had a greater probability of survival compared to those who had received only standard DVT prophylactic dosing [10]. To investigate the outcomes of using VTE prophylaxis rather than no prophylaxis in people with COVID-19, Paranjpe et al. conducted a retrospective study that included 2,773 patients hospitalized with COVID-19, of whom 28% of the patients had received systemic anticoagulation. The in-hospital survival rate was noted to be better in intubated patients who were anticoagulated (71%) than those who did receive anticoagulation (37%). No statistical significance was noticed in bleeding events that occurred in both groups. The study reported that in patients hospitalized with COVID-19, the use of systemic anticoagulation may be associated with better outcomes [11].

The timing of initiation of anticoagulation in hospitalized patients with COVID is also important. To evaluate whether early initiation of prophylactic anticoagulation in hospitalized patients with COVID is associated with a reduction in mortality, Rentsch et al. performed a cohort study that included 4,297 patients with severe infection. Prophylactic anticoagulation was initiated within the first 24 hours of admission in 84% of these patients. The cumulative incidence of mortality at 30 days in patients who received anticoagulation within 24 hours of admission was 14.3% compared to 18.7% in patients who did not. The study findings suggested that early initiation of prophylactic anticoagulation in patients hospitalized with COVID is associated with reducing 30-day mortality without increased risk of severe bleeding [12].

The INSPIRATION Randomized Clinical Trial investigated the effects of intermediate dose versus standard dose prophylactic anticoagulation in patients with severe COVID-19. The study assigned 600 patients admitted to the ICU with severe COVID-19 to receive enoxaparin at an intermediate dose (1 mg/kg daily) or standard prophylactic dosing (40 mg daily). The primary outcome was a composite of venous or arterial thrombosis, use of extracorporeal membrane oxygenation, or 30 days’ mortality. The primary outcome occurred in 45.7% in the intermediate dose prophylactic anticoagulation group and 44.1% in the standard dose prophylactic group with an odds ratio of 1.06. These findings suggested that the increase of the standard dosing to intermediate dosing did not improve the primary outcome. The 30-day mortality in both groups was also similar, and there was increased bleeding in the intermediate-dose group; however, there was no statistical significance [13].

To evaluate therapeutic anticoagulation compared with prophylactic anticoagulation, the ACTION trial assigned 615 patients hospitalized with COVID-19 with an elevated D-dimer to receive therapeutic dose anticoagulation (rivaroxaban 20 mg once daily in most cases) or prophylactic dose anticoagulation (prophylactic dosing of low molecular weight heparin in most cases). The primary outcome was a composite of survival, duration of hospitalization, and duration of supplemental oxygen. The results showed no statistically significant difference in primary outcome between the therapeutic anticoagulation group and the prophylactic anticoagulation group. Moreover, the bleeding risk was higher in the therapeutic group [14].

Meizlish et al. conducted a retrospective study to evaluate the effect of intermediate dose anticoagulation and aspirin on in-hospital mortality in patients with COVID-19. The investigators included 2,785 hospitalized patients with COVID-19. The study findings showed a reduction of cumulative incidence of in-hospital death associated with intermediate dose anticoagulation compared to prophylactic dose. A reduction of cumulative incidence of in-hospital death was also noticed with patients who received in‐hospital aspirin compared to those who did not receive antiplatelet therapy [15].

As the evidence of the role of anticoagulation in treating COVID-19 continues to evolve, different guidelines have been published by multiple societies. The American Society of Hematology (ASH) published guidelines on the use of anticoagulation for stroke prophylaxis in patients with COVID-19 in February 2021. The multidisciplinary guideline panel, formed by ASH, addressed prophylactic anticoagulation in two groups of patients. The two groups included critically ill patients that required ICU level of care and moderately ill patients who were admitted to the inpatient wards. The panel recommended prophylactic dose anticoagulation over intermediate and therapeutic intensity anticoagulation due to the small absolute risk difference. The ASH guideline did not show a preference for any of the prophylactic agents [16]. It was determined that there was low certainty that therapeutic dose anticoagulation would decrease all-cause mortality [17]. In addition, there was concern that therapeutic dose anticoagulation would be associated with an increased risk of major bleeding [18]. The effect of the intermediate dose was noted to reduce the risk of pulmonary embolism and DVT in the critically ill group but with low evidence [19].

The International Society on Thrombosis and Haemostasis - Scientific and Standardization Committee (ISTH-SSC) guidance in hospitalized patients with COVID-19 offered similar recommendations regarding the universal use of prophylaxis dose but also considered intermediate dose in high-risk critically ill patients. The ISTH-SSC also endorsed the combined use of pharmacological and mechanical thromboprophylaxis in critically ill patients [20]. The Anticoagulation Forum’s recommendations differed from the ASH and the American College of Chest Physicians (ACCP) recommendations regarding critically ill patients. The Anticoagulation Forum recommended a higher intermediate dose of thromboprophylaxis (e.g., enoxaparin 40 mg subcutaneous twice daily, enoxaparin 0.5 mg/kg subcutaneous twice daily, or heparin 7,500 units subcutaneous three times daily) [21]. These recommendations were based largely on expert opinion [22]. Also, the Anticoagulation Forum recommended the combined use of pharmacological and mechanical prophylaxis in critically ill patients. The Anticoagulation Forum recommended an individualized approach to post-discharge extended pharmacological prophylaxis with direct oral anticoagulants (DOACs). The Anticoagulation Forum also recommended against the daily monitoring of D-dimer level to guide anticoagulation dosing.

The American College of Cardiology recommended that hospitalized patients with COVID-19 should receive prophylactic-intensity anticoagulation unless contraindicated, such as those with disseminated intravascular coagulopathy with overt bleeding [23]. The 2020 CHEST COVID-19 guidelines recommended the use of standard dose anticoagulation for both acutely ill and critically ill patients, with emphasis on low molecular weight heparin to minimize exposure of the staff unless contraindicated as in acute kidney injury. The CHEST guidelines do not recommend the combined use of mechanical and pharmacological prophylaxis. The CHEST guidelines recommended against the use of intermediate or therapeutic dose anticoagulation but for treatment of proximal DVT and PE, with the latter for a minimum of three months. The CHEST guidelines do not recommend extended prophylaxis post-discharge. Additionally, the CHEST guidelines expressed concern for the possible interactions between DOACs and other medications that may harm the patient [24].

## Conclusions

Studies have shown that COVID-19 causes the body to enter a state of increased hypercoagulability. This increase in hypercoagulability may result in devastating consequences on the body such as VTE, organ failure, and death. Multiple studies have been conducted to determine the optimal treatment for hypercoagulability in patients with COVID-19; however, an optimal treatment protocol for hypercoagulation in COVID-19 remains undefined. Our study allows us to add to the complex information on anticoagulation indications and their role in changing primary end points in patient outcomes in the hospital. While several studies and guidelines suggest prophylactic dosing as most preferred in hospitalized patients with minimal risk factors, our study showed that patients who had received intermediate dosing of anticoagulation were found to have reduced thrombotic events, less requirement of high-flow nasal cannula and invasive mechanical ventilation, and shorter hospital lengths of stay. As the pandemic continues to hamper the American economy and slow forward mobility of healthcare and finance, it is crucial to study methods that not only upgrade clinical patient outcomes but also aid in tackling the heavy consequences of suffering a global pandemic. Though further randomized trials and prospective studies may be needed to reaffirm the results of this study, it still provides a satisfactory link between intermediate dosed anticoagulation and favorable outcomes.
